# Radiation-induced pulmonary gene expression changes are attenuated by the CTGF antibody Pamrevlumab

**DOI:** 10.1186/s12931-018-0720-4

**Published:** 2018-01-18

**Authors:** Mark D. Sternlicht, Ute Wirkner, Sebastian Bickelhaupt, Ramon Lopez Perez, Alexandra Tietz, Kenneth E. Lipson, Todd W. Seeley, Peter E. Huber

**Affiliations:** 1 FibroGen, Inc., San Francisco, USA; 2 Department of Translational Radiooncology, German Cancer Research Center (DKFZ), Heidelberg, Germany; 3 Department of Radiology, DKFZ, Heidelberg, Germany; 4 Department of Molecular and Radiation Oncology, DKFZ, Heidelberg, Germany; 5 Department of Radiation Oncology, University Hospital Center, Heidelberg, Germany

**Keywords:** CTGF, Radiation injury, Pulmonary fibrosis, Innate immune cells, Microarrays

## Abstract

**Background:**

Fibrosis is a delayed side effect of radiation therapy (RT). Connective tissue growth factor (CTGF) promotes the development of fibrosis in multiple settings, including pulmonary radiation injury.

**Methods:**

To better understand the cellular interactions involved in RT-induced lung injury and the role of CTGF in these responses, microarray expression profiling was performed on lungs of irradiated and non-irradiated mice, including mice treated with the anti-CTGF antibody pamrevlumab (FG-3019). Between group comparisons (Welch’s t-tests) and principal components analyses were performed in Genespring.

**Results:**

At the mRNA level, the ability of pamrevlumab to prolong survival and ameliorate RT-induced radiologic, histologic and functional lung deficits was correlated with the reversal of a clear enrichment in mast cell, macrophage, dendritic cell and mesenchymal gene signatures. Cytokine, growth factor and matrix remodeling genes that are likely to contribute to RT pneumonitis and fibrosis were elevated by RT and attenuated by pamrevlumab, and likely contribute to the cross-talk between enriched cell-types in injured lung.

**Conclusions:**

CTGF inhibition had a normalizing effect on select cell-types, including immune cells not typically regarded as being regulated by CTGF. These results suggest that interactions between RT-recruited cell-types are critical to maintaining the injured state; that CTGF plays a key role in this process; and that pamrevlumab can ameliorate RT-induced lung injury in mice and may provide therapeutic benefit in other immune and fibrotic disorders.

**Electronic supplementary material:**

The online version of this article (10.1186/s12931-018-0720-4) contains supplementary material, which is available to authorized users.

## Background

Radiation (RT) pneumonitis and fibrosis are side-effects that limit the utility of radiotherapy for thoracic cancers [1]. The mechanisms behind these responses are inadequately understood, and efforts to avoid or ameliorate them have seen little success.

Connective tissue growth factor (CTGF) is a key matricellular mediator of tissue remodeling and fibrosis in RT-injured lungs [2, 3]. In mice, CTGF inhibition was sufficient to inhibit lung remodeling due to RT, bleomycin and hyperoxia [3–6]. Increased CTGF is associated with lung fibrosis susceptibility [7–10], and its forced overexpression sensitized fibrosis-resistant mice to bleomycin-induced lung fibrosis [11]. CTGF is also overexpressed in injured lungs, and its targeted overexpression in fibroblasts elicited fibrosis in lung and other organs without addition of an injurious agent [12]. These and other results suggest common elements in fibrosis progression in these models and that CTGF neutralization may inhibit fibrosis. Nevertheless, precise mechanisms whereby CTGF contributes to fibrotic disease are not entirely understood.

In humans, CTGF levels are elevated in patients with fibrotic lung disease, including idiopathic pulmonary fibrosis (IPF), bronchopulmonary dysplasia, sarcoidosis, and systemic sclerosis [6, 13–18]. Thus, CTGF participates broadly in lung fibrosis, and its inhibition may be beneficial in various fibrotic diseases. Accordingly, a human anti-CTGF monoclonal antibody, pamrevlumab (FG-3019), is currently undergoing clinical testing in IPF and other indications.

In this study, microarray expression profiling was used to examine the role of CTGF in a murine model of RT-induced lung injury. The evolution of lung injury in murine RT models resembles that of human RT injury, with RT exposure causing acute free-radical tissue damage (days), pneumonitis (2–16 weeks) and subsequent development of pulmonary fibrosis. To evaluate the role of CTGF in radiation-induced lung injury [3], pamrevlumab was administered for 8 weeks beginning 2 days before or 2, 20 or 112 days after a single dose of thoracic RT. As described elsewhere [3], pamrevlumab treatment prolonged overall animal survival and prevented and reversed pulmonary remodeling as indicated by computed tomography (CT), histology and blood gas analysis.

Here, we report an in depth analysis of gene expression changes in mouse lungs from a previously published experiment [3]. Novel observations include pamrevlumab treatment-elicited, schedule-dependent amelioration of an RT-associated expression pattern highly enriched in mast cell, macrophage, dendritic cell and mesenchymal transcripts. At 18 weeks, the effect of pamrevlumab was consistent with prior histologic and CT observations, with the RT-induced expression pattern being almost entirely normalized by pamrevlumab treatments begun at 20 or 112 days, i.e., as little as 2 weeks prior to sacrifice in the 112-day group, whereas regimens initiated 2 days before or after RT had little effect on the RT-induced expression pattern. At 30 weeks, a similar but diminished RT response pattern was essentially normalized by all pamrevlumab regimens, suggesting an accelerated resolution of immune and fibrogenic processes after 18 weeks.

These changes provide insight into the signaling networks that likely regulate RT-induced lung injury and suggest that CTGF influences mesenchymal cells, as well as select immune cell-types that are not usually recognized as being CTGF-responsive. We propose a model in which CTGF inhibition results in a synchronized reprogramming of multiple interdependent cellular programs; replacing programs that contribute to tissue damage or fibrosis with programs that promote organ repair.

## Methods

### RT and Pamrevlumab administration

**Fig. 1:**
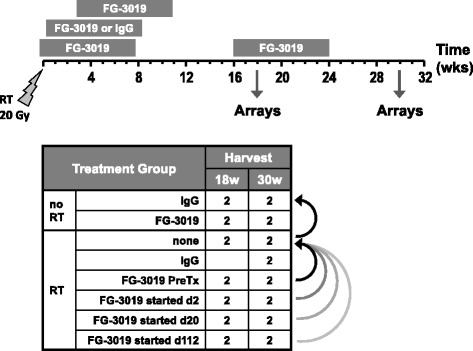
Study design. Eight-week pamrevlumab (FG-3019) treatment regimens were initiated before or after thoracic RT. Lungs were harvested at 18 or 30 weeks for expression profiling. Two biologic replicate arrays were analyzed per treatment group at each time-point, and were compared as indicated (curved arrows)

Animal procedures were approved by institutional and governmental authorities and are detailed elsewhere [3]. Female C57BL/6J mice were anesthetized and a single thoracic 20 Gy RT dose was delivered by linear accelerator. Pamrevlumab (FibroGen) was administered i.p. at 10 mg/kg TIW for 8 weeks starting 2 days before or 2, 20 or 112 days after RT (Fig. 1). Polyclonal human IgG (Sigma) was similarly administered beginning 2 days after RT. Pamrevlumab and IgG were similarly administered to non-irradiated mice beginning 2 days after RT was delivered to contemporaneously irradiated mice. Two mice per group were sacrificed 18 and 30 weeks after RT for expression profiling.

### Expression analysis

RNA from nitrogen-frozen lower right lung lobes was profiled on 4x44K whole genome arrays (Agilent G4122F) and analyzed using Agilent GeneSpring GX software. Chip data were normalized to the 50th percentile of all measurements, probe data were normalized to the median of contemporaneous non-irradiated controls, and low intensity probes with raw signal <3X the cross-gene error model coefficient on ≥28 arrays were removed. Altered probes were defined as having a > 2-fold difference in expression at *p* < 0.05 (t-test). Major expression patterns of probes altered at 18 or 30 weeks by RT vs. non-irradiated IgG controls or by pamrevlumab vs. RT alone were further identified by principal components analysis. Coordinately altered probes were correlated with principal component 1 at *p* < 0.001. Data are available at http://www.ebi.ac.uk/arrayexpress/experiments/E-TABM-1153/.

Gene Ontology (GO) analyses were performed using GOrilla [19](http://cbl-gorilla.cs.technion.ac.il/). Pertinent GO terms were defined as having ≥9 unique genes, >3X as many members as expected in an altered list (*p* < 0.001), and no further sub-categories meeting these criteria. Functional relationships between PCA1-ordered genes were investigated by gene-set enrichment analysis (GSEA) [20]. Leading edge analysis was performed on gene-sets with a family-wise or nominal *p* < 0.01. Regulatory interactions between altered genes were identified using Pathway Studio MammalPlus 11.4.0.8 [21].

To investigate changes in cellular content, lists of cell-type distinguishing genes were derived using the 182-array Novartis C57Bl/6 GeneAtlas (GSE10246) and a 120-array human dataset for additional cell-types (Additional file 1: Table S1). Cell-type distinguishing transcripts were defined as having average expression >64X higher in a given cell-type vs. all other samples (nonparametric *p* < 0.001), with genes on > 1 list being removed. To assess the behavior of these genes in our dataset, Affymetrix gene lists (Additional file 2: Table S2) were linked to probes on the Agilent platform.

## Results

### Pamrevlumab normalizes RT-induced gene expression changes

To explore the role of CTGF in RT injury, we performed microarray-based expression profiling on lung samples from a previously published study [3] that were obtained 18 and 30 weeks after a single thoracic RT dose, with the anti-CTGF antibody pamrevlumab being administered for 8 weeks beginning 2 days before or 2, 20 or 112 days after RT (Fig. 1). The origin of these samples and their relevance to functional outcomes is detailed elsewhere [3]. Microarray probes associated with substantial and significant gene expression changes (> 2-fold*, p* < 0.05) were identified and lists of genes altered by RT (RT alone vs. non-irradiated IgG controls, 2570 probes) or by pamrevlumab in the background of RT (any pamrevlumab treatment vs. RT alone, 3644 probes) were combined (4798 unique probes total).

**Fig. 2:**
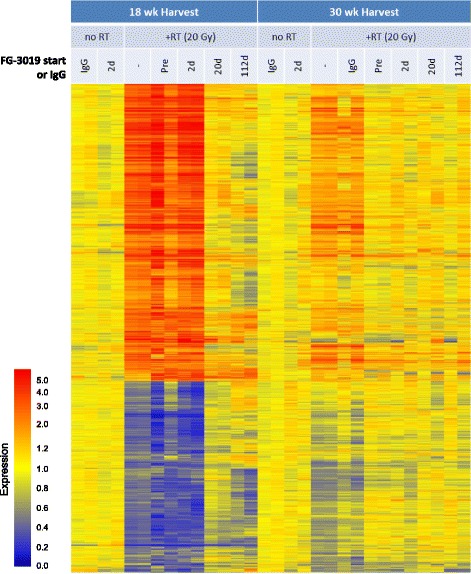
Hierarchical clustering of regulated RT and pamrevlumab (FG-3019) responsive genes (*n* = 2754). Expression levels are normalized to the median of time-matched non-irradiated controls, with relative increases and decreases being indicated in red and blue, respectively, and neutral changes indicated in yellow, as indicated by the adjacent relative expression scale

Shared expression patterns were identified from this combined list by principal components analysis. The predominant pattern (PCA1) accounted for 68% of the total variance, and exhibited a robust RT response at 18 weeks that was almost entirely attenuated by pamrevlumab treatment begun 20 or 112 days after RT, but not by earlier treatments. PCA1 was also characterized by a milder RT response at 30 weeks that was largely attenuated by all pamrevlumab regimens. A refined set of genes coordinately regulated with respect to this pattern (i.e., correlated with PCA1 at *p* < 0.001) (Additional file 3: Table S3) was investigated by hierarchical clustering (Fig. 2). This set of 2754 coordinately regulated probes (hereafter referred to as RT and pamrevlumab responsive genes) was easily divided into probes that were elevated by RT and reversed by pamrevlumab (1676 probes, 1344 named genes, hereafter referred to as RT-elevated genes, with the understanding that their RT-mediated elevation was also reversed by pamrevlumab) and probes that were diminished by RT and elevated by pamrevlumab (1078 probes, 769 named genes, hereafter referred to as RT-diminished genes). Probes meeting change criteria but not sharing the above pattern were omitted, as they tended to exhibit low or hypervariable fluorescence with no obvious patterns linked to time or treatment.

At 18 weeks, resolution of the RT response was evident after pamrevlumab treatment initiated 20 or 112 days after RT, with resolution being apparent after only 2 weeks for the regimen initiated on day 112. At 30 weeks, RT-response genes showed a tendency to be attenuated by all pamrevlumab regimens. In contrast, pamrevlumab did not affect expression in non-irradiated mice, with only three probes meeting change criteria at both 18 and 30 weeks, but with hypervariable expression inconsistent with a true treatment response.

### Many RT and pamrevlumab response genes are immune cell related

To investigate functional relationships between coordinately altered genes, GO analyses were performed. The majority of GO categories associated with the RT-elevated gene-set were immune cell-related, with “immune system process” (GO:0002376) exhibiting the greatest over-representation among RT-elevated genes (3.5-fold, *p* = 1e-50).

To obtain greater specificity, 108 GO categories were identified with ≥9 total members, > 3-fold enrichment in the RT-elevated gene-set at *p* < 0.001, and no further child sub-categories. The majority of these GO terms were immune cell-related, including categories concerning cytokines, chemokines, lymphocyte regulation, antigen-presenting cells and neutrophils (Additional file 4: Table S4). Other RT-elevated categories concerned extracellular matrix (ECM), integrins, tissue remodeling, or cell division. Thus, a substantial fraction of RT-elevated genes were immune response related, whereas an immune cell source seems unlikely for some RT response genes, including ECM genes. A smaller number of GO categories associated with the RT-diminished gene-set were related to muscle cell contraction, and drug, retinol or steroid metabolism (Additional file 5: Table S5).

Immune cell enrichment was also suggested by GSEA (Additional file 6: Table S6). The gene-set with the highest enrichment score was associated with foamy macrophage enrichment in lung [22]. Leading edge analysis of enriched gene-sets revealed three major clusters of shared ECM, cytokine and cell cycle genes, qualitatively validating our GO results. A coordinated pattern of RT-diminished and pamrevlumab-resolved glutathione metabolism genes was also identified.

### Enrichment of mesenchymal and immune cell distinguishing genes in RT-injured lungs

To objectively score changes in cellular content, we analyzed public expression datasets to identify genes that could distinguish specific cell-types, using both the Novartis mouse GeneAtlas and a human atlas compiled to include cell-types absent in the Novartis dataset (Additional file 1: Table S1). By identifying genes with expression > 64-fold higher in a given cell-type vs. other cell-types (Wilcoxon-Mann-Whitney *p* < 0.001), we compiled non-overlapping cell-type distinguishing gene lists for cell-types of interest (Additional file 2: Table S2). The degree to which these genes were over- or under-represented in the RT-elevated gene-set was then determined.

As indicated in Table 1, macrophage genes were highly enriched in the RT-elevated gene-set (14-fold, χ^2^ *p* <  0.0001). Mast cell, dendritic cell and mesenchymal cell genes were also seen 5- to 10-times more often than expected in the RT-elevated gene-set (*p* <  0.0001), whereas neutrophil, B-cell and endothelial genes were mildly over-represented. T-cell and lung epithelial genes did not appear enriched, and retinal epithelial genes included as a negative control were under-represented in the RT-elevated gene-set. These results suggest that RT causes a proportional enrichment of select cell-types in lung, most notably macrophages, mast cells, dendritic cells and mesenchymal cells, and that these effects can be reversed by pamrevlumab treatment.

**Table 1:** Proportional representation of cell-type distinguishing genes among RT-elevated genes

Cell Type	(n, N)	Fold Enrichment	*p*
Macrophage	(98, 123)	14.2	3E-148
Dendritic Cell	(26, 45)	10.3	6E-32
Mesenchymal Cell	(28, 73)	6.8	1E-23
Mast Cell	(42, 149)	5.0	8E-25
Neutrophil	(14, 49)	3.3	2E-05
B Cell	(11, 81)	2.4	0.005
Endothelial Cell	(9, 43)	2.4	0.012
T Cell	(7, 81)	1.5	0.27
Epithelial Cell (Lung)	(10, 91)	1.3	0.46
Epithelial Cell (Retina)	(2, 115)	0.31	0.08

Enrichment = (n/t)/(N/T), where n = cell-type distinguishing probes in RT-elevated gene set, N = total cell-type distinguishing probes, t = total RT-elevated probes = 1676, and T = total Agilent probes on array platforms from which neutrophil, endothelial or lung epithelial genes were identified (*T* = 19,515) or from which all other cell-type distinguishing genes were identified (*T* = 29,806). Observed proportions of cell-type distinguishing genes in the RT-elevated gene set were compared to the proportions expected by chance using the χ^2^ statistic

Fold-changes in expression of cell-type distinguishing transcripts were also consistent with enrichment of specific cell-types. The most highly RT-elevated genes (Table 2) included five mast cell protease genes elevated 23-to-110-fold by RT at 18 weeks. *Mmp12*, a macrophage gene, was also elevated 24-fold at 18 weeks, while other macrophage transcripts were increased 6-to-7-fold. Examples of profoundly regulated genes defining a given cell-type among lesser regulated genes may suggest that specific transcripts are subject to additional regulation beyond simple changes in cell-type representation. Nevertheless, using panels of genes to infer changes in cellular representation represents a relatively comprehensive and systematic approach as compared to relying on individual markers that may be misclassified as cell-type archetypes, subject to other forms of regulation, or difficult to score in some tissues.

**Table 2:** Cell-Type Distinguishing Genes Most Highly Elevated by RT

		18w Fc vs noRT		30w Fc vs noRT	
Gene ID	Description	RT	RT + FG	RT	RT + FG
Mast Cell					
Mcpt4	mast cell protease 4	109.9	5.9	10.7	2.2
Cpa3	carboxypeptidase A3, mast cell	94.2	3.3	19.9	3.0
Cma2	chymase 2, mast cell protease 9	51.6	3.6	9.0	2.0
Cma1	chymase 1, mast cell	46.5	2.7	4.8	1.1
Tpsg1	tryptase gamma 1, mast cell protease 2	22.7	2.7	40.2	5.0
Mrgprb1	MAS-related GPR, member B1	20.3	2.9	8.5	1.7
Darc	Duffy blood group, chemokine receptor	18.5	1.9	2.3	0.9
Tpsb2	tryptase beta 2, mast cell protease 6	18.0	2.5	5.5	3.8
Macrophage					
Mmp12	matrix metallopeptidase 12	24.0	0.4	6.0	1.2
Trem2	triggering receptor expressed on myeloid cells 2	15.5	1.4	7.0	1.6
Msr1	macrophage scavenger receptor 1	10.0	0.5	5.1	1.2
Cd200r1	CD200 receptor 1	7.1	1.0	3.1	1.1
Ccl3	chemokine ligand 3	6.9	0.8	1.3	0.4
Ms4a7	membrane-spanning 4-domains, subfamily A, member 7	6.8	1.0	2.5	0.9
Fcgr2b	Fc receptor, IgG, low affinity IIb	6.6	0.7	2.2	1.3
Ms4a6d	membrane-spanning 4-domains, subfamily A, member 6D	6.5	0.4	2.4	1.2
Dendritic Cell					
Cxcl9	chemokine (C-X-C motif) ligand 9	79.8	4.0	1.1	0.5
Kynu	kynureninase	6.5	0.8	2.3	1.3
Dnase1l3	deoxyribonuclease 1-like 3	5.6	2.5	0.9	1.0
Serpina3g	serine peptidase inhibitor, clade A, member 3G	5.3	1.1	1.6	1.1
Ppfia4	protein tyrosine phosphatase, f polypeptide, interacting, a4	5.1	1.3	2.5	1.3
Ffar2	free fatty acid receptor 2	4.1	1.8	1.8	0.9
Itgax	integrin alpha X	4.1	0.7	1.9	0.8
Ccl5	chemokine (C-C motif) ligand 5	3.9	1.7	0.9	0.8
Mesenchymal Cell					
Dlk1	delta-like 1 homolog (Drosophila)	15.2	4.7	26.3	10.4
Timp1	tissue inhibitor of metalloproteinase 1	8.2	0.4	3.6	0.8
Fn1	fibronectin 1	7.4	1.3	3.9	1.7
Wisp1	WNT1 inducible signaling pathway protein 1	5.3	0.9	2.4	1.5
Ptx3	pentraxin related gene	4.7	1.1	1.6	1.2
Col5a2	collagen, type V, alpha 2	4.5	0.6	1.7	1.1
Wisp2	WNT1 inducible signaling pathway protein 2	4.0	0.9	2.7	1.3
Fstl1	follistatin-like 1	4.0	1.0	2.4	1.3
Neutrophil					
Clec7a	C-type lectin domain family 7, member a	6.0	0.7	1.5	0.6
Aif1	allograft inflammatory factor 1	4.1	1.1	2.0	1.1
Ffar2	free fatty acid receptor 2	4.1	1.8	1.8	0.9
P2ry13	purinergic receptor P2Y, G-protein coupled 13	2.7	0.4	1.3	0.9
Fpr1	formyl peptide receptor 1	2.4	0.7	1.6	0.8
Lst1	leukocyte specific transcript 1	2.3	0.8	1.1	0.8
Mnda	myeloid cell nuclear differentiation antigen	2.1	1.5	1.5	1.1
B-cell					
Igl-V1	immunoglobulin lambda chain (IgL) mRNA	8.0	1.2	0.7	1.4
March1	membrane-associated ring finger (C3HC4) 1	3.3	1.1	1.7	1.3
Ulbp1	UL16 binding protein 1	3.3	0.7	1.8	1.3
Cd22	CD22 antigen	3.0	2.4	1.3	1.3
H2-DMb2	histocompatibility 2, class II, locus Mb2	2.8	1.1	0.9	1.3
Tlr1	toll-like receptor 1	2.7	1.0	1.1	1.1
Mef2c	myocyte enhancer factor 2C	2.2	1.7	1.5	0.9
Endothelial Cell					
Col4a1	collagen, type IV, alpha 1	3.20	0.6	1.82	1.2
Col4a2	collagen, type IV, alpha 2	3.11	0.5	1.76	1.2
Fabp4	fatty acid binding protein 4, adipocyte	3.01	1.2	1.73	0.5
Mgp	matrix Gla protein	2.07	0.7	1.39	1.1
Multiple Potential Cell Sources					
Ankrd34b	ankyrin repeat domain 34B	224.3	8.9	8.9	1.1
Chl1	cell adhesion molecule with homology to L1CAM	93.8	1.3	15.6	1.0
Ucma	upper zone of growth plate and cartilage matrix associated	71.2	1.8	9.9	1.2
Tnfsf18	tumor necrosis factor (ligand) superfamily, member 18	46.8	3.6	2.8	1.0
Gdf3	growth differentiation factor 3	32.1	3.9	4.2	1.1
Ear5	eosinophil-associated, ribonuclease A family, member 5	29.5	2.1	18.3	1.6
Ccl8	chemokine (C-C motif) ligand 8	29.0	2.0	5.4	0.9
Dio3	deiodinase, iodothyronine type III	25.6	1.4	10.3	1.1

Fold-change (Fc) values are relative to unirradiated (noRT) controls for RT alone and RT + FG-3019 (FG) treatment begun 16 weeks after RT

Genes with exemplary fold-change values that were not distinguishing for a specific cell-type by our criteria may still reflect or contribute to changes in cell-type representation. For example, the gene most highly elevated by RT, *Ankrd34b*, influences promyeloid progenitor cell induction [23]. Thus, *Ankrd34b* may be expressed by and contribute to enrichment of mast cells, dendritic cells and macrophages in irradiated lung. Likewise, many chemokines strongly elevated by RT (Table 3) were not scored as cell-type distinguishing, as they can arise from multiple myeloid cell-types.

**Table 3:** Cytokines and Growth Factors Altered by RT and FG-3019

		18w Fc vs noRT		30w Fc vs noRT	
Gene ID	Description	RT	RT + FG	RT	RT + FG
Chemokines (Chemotaxis)					
Cxcl9	chemokine (C-X-C motif) ligand 9	79.8	4.0	1.1	0.5
Ccl8	chemokine (C-C motif) ligand 8 (MCP-2)	29.0	2.0	5.4	0.9
Cxcl10	chemokine (C-X-C motif) ligand 10	17.2	1.5	1.0	0.5
Ccl1	chemokine (C-C motif) ligand 1	8.9	2.1	2.9	1.2
Ccl12	chemokine (C-C motif) ligand 12 (MCP-5)	8.3	0.6	1.9	0.7
Ccl7	chemokine (C-C motif) ligand 7 (MCP-3)	7.9	0.6	1.3	0.5
Ccl2	chemokine (C-C motif) ligand 2 (MCP-1)	7.3	0.6	1.1	0.5
Ccl3	chemokine (C-C motif) ligand 3 (MIP-1a)	6.9	0.8	1.3	0.4
Ccl6	chemokine (C-C motif) ligand 6	5.8	0.7	2.8	1.1
Ccl9	chemokine (C-C motif) ligand 9 (MIP-1 g)	5.7	0.8	2.2	1.0
Ccl5	chemokine (C-C motif) ligand 5 (RANTES)	3.9	1.7	0.9	0.8
Ccl17	chemokine (C-C motif) ligand 17	3.7	1.1	0.9	0.7
Ccl4	chemokine (C-C motif) ligand 4 (MIP-1 p)	3.6	1.4	0.9	0.6
Cxcl1	chemokine (C-X-C motif) ligand 1	2.4	0.6	0.7	0.5
Cxcl16	chemokine (C-X-C motif) ligand 16	2.3	0.7	1.4	0.9
Cxcl12	chemokine (C-X-C motif) ligand 12 (SDF-1)	2.2	0.5	1.4	1.1
Cxcl3	chemokine (C-X-C motif) ligand 3 (MIP-2p)	2.0	0.5	2.2	0.7
Cytokines (Cell Activation)					
Tnfsf18	tumor necrosis factor (ligand) superfamily, member 18	46.8	3.6	2.8	1.0
Il1rn	interleukin 1 receptor antagonist	5.7	1.2	1.8	0.7
Il6	interleukin 6	4.7	1.0	1.7	0.6
Il12b	interleukin 12b	4.7	1.6	0.9	0.8
Tnfsf8	tumor necrosis factor (ligand) superfamily, member 8	4.4	1.6	1.9	1.1
C1qtnf9	C1q and tumor necrosis factor related protein 9	3.8	1.7	2.6	1.2
C1qtnf5	C1q and tumor necrosis factor related protein 5	2.5	1.0	1.7	1.2
Il18	interleukin 18	2.4	0.6	1.6	1.0
Il4	interleukin 4	2.4	1.7	0.9	0.7
Il12a	interleukin 12a	0.5	1.1	0.6	1.4
TGFβ / BMP Signaling (Differentiation, Migration, ECM Production,...)					
Gdf3	growth differentiation factor 3	32.1	3.9	4.2	1.1
Inhba	inhibin beta-A	7.8	1.1	1.9	1.2
Gdf6	growth differentiation factor 6 (BMP13)	5.9	0.6	3.7	1.8
Fst	follistatin	5.7	0.8	1.5	1.1
Grem1	gremlin 1	4.7	1.8	1.5	0.8
Gdf15	growth differentiation factor 15	4.0	1.7	3.0	2.6
Fstl1	follistatin-like 1	4.0	1.0	2.4	1.3
Ltbp2	latent transforming growth factor beta binding protein 2	3.9	1.0	2.1	1.3
Ctgf	connective tissue growth factor	3.5	1.1	1.2	0.8
Bmp8a	bone morphogenetic protein 8a	2.8	1.2	1.9	1.5
Bmp6	bone morphogenetic protein 6	0.2	0.3	0.4	0.7
Wnt Signaling (Cell Polarity, Pattern Formation)					
Wnt10a	wingless related MMTV integration site 10a	22.1	1.1	2.3	1.1
Frzb	frizzled-related protein	8.5	1.2	2.2	0.9
Sfrp1	secreted frizzled-related protein 1	6.3	0.6	3.2	1.0
Wisp1	WNT1 inducible signaling pathway protein 1	5.3	0.9	2.4	1.5
Wisp2	WNT1 inducible signaling pathway protein 2	4.0	0.9	2.7	1.3
Fzd2	frizzled homolog 2 (Drosophila)	2.8	0.9	1.8	1.2
Wnt7a	wingless-related MMTV integration site 7A	2.7	1.2	1.3	0.9
Fzd7	frizzled homolog 7 (Drosophila)	2.0	0.8	1.5	0.8
IGF Signaling (Proliferation, Survival)					
Insl6	insulin-like 6	7.9	1.4	3.1	1.2
Igf1	insulin-like growth factor 1	6.3	0.7	4.7	1.3
Igfbp2	insulin-like growth factor binding protein 2	3.5	0.8	1.9	1.6
Igfbp4	insulin-like growth factor binding protein 4	2.6	1.4	1.5	1.1
Igfbp7	insulin-like growth factor binding protein 7	2.5	0.7	1.5	1.2
Igfbp6	insulin-like growth factor binding protein 6	0.3	0.6	0.6	0.9
Other Cytokines & Growth Factors					
Retnla	resistin like alpha	10.5	1.7	6.4	1.4
Angptl7	angiopoietin-like 7	5.0	1.4	1.0	1.0
Pdgfc	platelet-derived growth factor, C polypeptide	4.2	0.4	1.5	1.0
Csf3	colony stimulating factor 3 (G-CSF)	3.3	2.3	1.4	1.1
Angptl3	angiopoietin-like 3	3.2	1.2	1.8	1.3
Fgf2	fibroblast growth factor 2	2.5	1.3	1.4	0.9
Pdgfd	platelet-derived growth factor, D polypeptide	2.2	0.9	1.9	1.4
Pgf	placental growth factor	2.1	0.9	1.1	0.8
Fgfbp1	fibroblast growth factor binding protein 1	0.4	0.6	0.7	1.1
Cytl1	cytokine-like 1	0.3	1.0	0.4	0.7
Retn	resistin	0.2	1.2	0.2	0.3

Fold-change (Fc) values are relative to unirradiated (noRT) controls for RT alone and RT + FG-3019 (FG) treatment begun 16 weeks after RT

While many RT-elevated genes were tentatively linked to changes in representation of specific cell-types, changes in other genes may reflect the net effect of changes in multiple cell-types. For example, the RT-mediated induction of *Spp1* may reflect an increased abundance or activation of macrophages and/or mesenchymal cells, as it is highly expressed by both cell-types. Likewise, *Ctgf* was most highly expressed by two key cell-types in our atlases, mesenchymal cells and endothelial cells, less consistently expressed in lung and other epithelial cell-types, and essentially absent from hematopoietic lineage cell-types (Additional file 1: Table S1). Thus, the 3.5-fold increase in *Ctgf* expression 18 weeks after RT and its resolution by pamrevlumab treatments (Fig. 4) may reflect changes in the content or activation of multiple cell-types, including type II alveolar epithelial cells [14]. However, of the cell-types that were highly enriched in the RT-elevated gene-set, only mesenchymal cells express CTGF, consistent with the idea that these cells are likely to be largely responsible for the increase in *Ctgf* expression following RT.

Since macrophages display classical (M1) and alternative (M2) activation phenotypes, and since M2 macrophages promote fibrosis [24], we further characterized the macrophage-associated transcripts in our dataset. Prototypic M2 markers *Arg1*, *Chi3l3*, *Chi3l4*, *Retnla* and *Mrc1* [24] were elevated 3- to 10-fold by RT at 18 weeks and 2- to 6-fold at 30 weeks, whereas M1 markers, such as *Nos2*, *Tnf*, *Il16*, *Il12a* and *Il1b* [25], were not altered by RT or pamrevlumab. These data suggest that M2 macrophages are substantially enriched in RT-injured lung and that pamrevlumab decreases M2 activation in irradiated lung.

### Kinetic resolution of RT-induced changes and Pamrevlumab effects

**Fig. 3:**
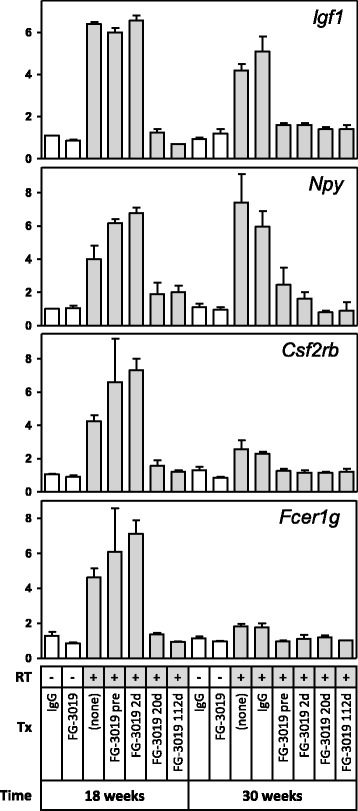
Differential resolution of RT-induced gene expression over time. Igf1 and Npy are likely to be macrophage transcripts, Csf2rb is likely to be a mast cell transcript, and Fcer1g can be expressed by both of these cell-types. Relative expression is normalized to contemporaneous non-irradiated controls (mean ± SE)

Differences in the kinetics of gene expression were also evident, with the level of RT induction for most genes declining between 18 and 30 weeks (Fig. 2). Few genes, including macrophage genes *Igf1* and *Npy*, retained a high level of RT induction at 30 weeks, although they still resembled other RT-regulated transcripts as being attenuated by pamrevlumab treatment (Fig. 3). More commonly, macrophage genes showed a decline in RT-induced expression over time to baseline (e.g., *Csf2rb* and *Fcer1g*). It was not possible to discriminate whether the differential resolution of cell-type distinguishing genes reflected changes in the proportional representation of cell-types expressing those particular genes and/or changes in transcriptional activity. In general, however, patterns observed at 18 weeks were often evident at 30 weeks, but less pronounced. At 30 weeks, mast cell transcripts also exhibited the greatest RT-induced increase, with macrophage genes again showing a milder degree of elevation and other cell-types displaying few substantially altered genes (Table 2).

**Fig. 4:**
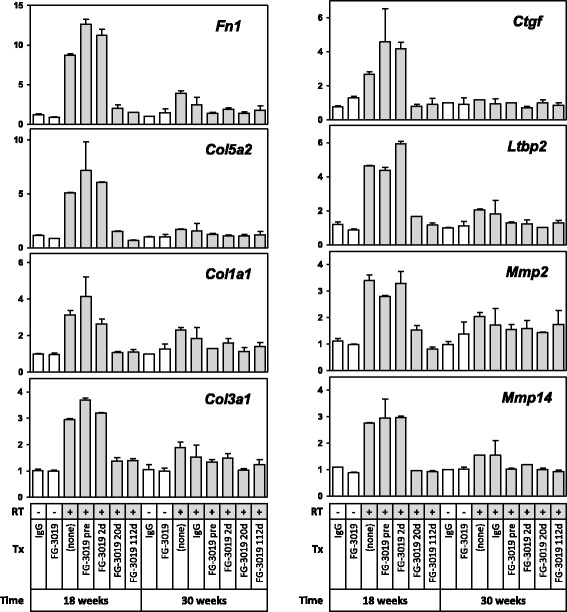
Relative expression profiles of selected mesenchymal, ECM remodeling genes in response to RT and pamrevlumab (FG-3019) at 18 and 30 weeks. Relative expression is normalized to contemporaneous non-irradiated controls (mean ± SE)

This attenuation of RT-altered genes at 30 weeks encompassed many mesenchymal cell derived ECM remodeling genes. *Ctgf,* which was elevated 3.5-fold by RT at 18 weeks, showed little if any elevation by 30 weeks with or without pamrevlumab treatment (Fig. 4). Thus, although fibrotic ECM deposits were histologically present at 30 weeks in untreated animals and in mice whose pamrevlumab administration began 2 days before or after irradiation, fibrogenic processes in RT-treated animals may have diminished together with immune processes by 30 weeks. With regards to pamrevlumab, the initiation of treatment 2 days before or after RT had little effect on gene expression at 18 weeks, whereas by 30 weeks, all pamrevlumab regimens appear to have reversed the RT-induced gene response. Thus, in accordance with prior CT and histologic findings, all treatments accelerated a trend towards normalization of gene expression over time, with the kinetics of reversal occurring very rapidly in the 112-day treatment group.

### Reciprocal signaling between enriched cell types

**Fig. 5:**
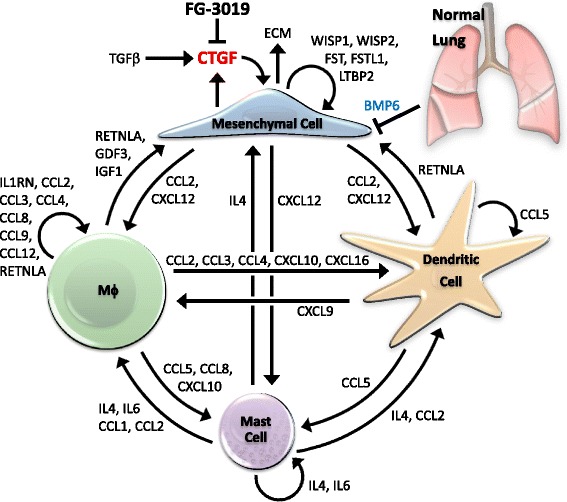
Cytokine and growth factor cross-talk in late RT-induced lung injury. The mRNAs enriched in response to RT and diminished by pamrevlumab (FG-3019) were used to develop a model of autocrine and paracrine cell-cell communication in RT injured lungs. The RT-induced decrease in endothelial cell-derived BMP6 expression is indicated by blue lettering. The model weights robustness of change, atlas- and literature-based evidence pertaining to proposed cell-type origins and targets, and literature associations with pulmonary fibrosis or other forms of fibrosis

The coordinated changes in cell-type distinguishing genes suggest there is interdependent communication between the RT-enriched cell-types. Regulatory links between the altered cytokines and growth factors were thus explored and likely cell-type origins inferred by inspection of our cell-type atlases and other public data. Functional roles for these factors include recruitment, proliferation, survival and/or activation of select cell-types. This analysis strongly suggested reciprocal signaling between the RT-enriched cell-types and lung cell-types involved in lung homeostasis, injury and repair (Fig. 5). From this analysis, we conclude that an RT-induced expression profile is maintained by CTGF and by complex cell-cell interactions, and that this pattern can be disrupted by pamrevlumab treatment.

Our analysis indicated that specific factors, such as mast cell-derived IL4 and IL6, macrophage-derived IGF1, mesenchymal CXCL12 and endothelial BMP6, have fairly certain cell origins. Regulatory interactions identified in Pathway Studio also highlight the potential for complex cross-talk between RT-altered factors [21]. Our analysis suggests that RT-induced CXCL12 from mesenchymal cells may promote the migration of mast cells, macrophages and dendritic cells into lung [26–28]. Mast cell-derived IL4 and IL6 can be induced by mesenchymal CXCL12, by macrophage-derived CCL3, and by CCL2 and IL18, which can come from multiple cell sources. In return, IL4 and IL6 can elicit each other’s expression, as well as IGF1, CCL3, CCL4, CCL7, CCL17, CXCL10, IL1RN and RETNLA in macrophages, CXCL9 in dendritic cells, and CCL2 and CCL5 in multiple cell-types. Conversely, macrophage-derived IL1RN can suppress IL6, CCL2 and CCL5 expression. Dendritic cell CXCL9 and macrophage CXCL10 can positively regulate one another, as can macrophage-derived CXCL10 and CCL3. CXCL10 and CCL3 can elicit CCL2 and CCL5 in multiple cell-types, while CCL2 and IL18 can elicit IL4 and IL6 in mast cells, CXCL10 and CCL3 in macrophages, and CCL5 in macrophages, dendritic cells and NK cells. CCL5, in turn, can elicit IL6, CCL2, CCL3, CCL4, CCL7 and CXCL10. In addition, FGF2 from mesenchymal cells can elicit mast cell IL6, macrophage IGF1, mesenchymal INHBA and IGFBP4, and CCL2 in multiple cell-types, as well as suppress endothelial BMP6, while at least three of these factors (IL6, IGF1 and INHBA) can induce CTGF expression. Thus, we recognize several potential regulatory circuits among the various factors regulated by RT and pamrevlumab. Moreover, many of these factors are also elevated in IPF, including CXCL12, IL4, CCL2, IL1RN, IGF1, and CTGF itself [8, 14–17, 28–32].

Pathway analyses also indicated that at least 20% of the RT-elevated genes are downstream targets of TGFβ, an established and potent inducer of CTGF, whereas the only other factors known to regulate > 20% of RT-elevated genes, tumor necrosis factor and interferon γ, have been shown to suppress CTGF expression [21]. Thus, although each of these factors contribute to RT-induced lung injury [33], interactions between CTGF and TGFβ are likely to be key drivers of the molecular changes seen in the current study [4].

## Discussion

CTGF is a key mediator of tissue fibrosis. While CTGF is produced by and affects mesenchymal cell-types [34], its effects on immune cell infiltrates that often accompany and contribute to fibrosis remain largely unexplored. In our RT injury model, CTGF mRNA enrichment occurred concurrently with a broader enrichment of mesenchymal transcripts. Interestingly, pamrevlumab-mediated inhibition of CTGF for as little as 2 weeks resulted in a profound normalization of RT-induced gene expression changes that largely appeared to reflect changes in mast cell, macrophage and dendritic cell infiltration. This provides novel insights into how CTGF inhibition may produce beneficial therapeutic effects. The interactions between CTGF, mesenchymal cells and immune cells described here extend correlative data indicating that CTGF is enriched in a wide variety of inflammatory disorders, and support a model in which CTGF regulates multiple interdependent cellular programs that dictate whether tissue maintenance, damage or repair take place.

### RT- and pamrevlumab-related changes in gene expression correlate with other indicators of pulmonary damage and repair

Expression data were consistent with previously reported histologic, radiologic and functional data indicating that pamrevlumab attenuated or reversed pulmonary damage caused by an otherwise lethal dose of RT [3]. At 18 weeks, there was near complete normalization of an RT-induced gene signature rich in immune cell transcripts by pamrevlumab treatments begun 20 or 112 days after RT exposure, but not by treatments begun 2 days before or after RT. This is in agreement with histologic data indicating that a profound increase in pulmonary leukocytes at 18 weeks was almost entirely attenuated by pamrevlumab begun 20 or 112 days after RT, but not 2 days before or after RT. Likewise, longitudinal CT imaging revealed progressive RT-induced increases in lung density that were attenuated at 18 weeks by pamrevlumab treatments begun at 20 or 112 days, but not immediately before or after RT exposure.

At 30 weeks, the gene response to RT was milder than at 18 weeks, and was attenuated by all four pamrevlumab regimens. It is unclear whether pamrevlumab treatment shortly before or after irradiation accelerated a natural resolution of the RT gene response or whether the extent of the response from which recovery began had been diminished by treatment so that baseline was reached more quickly. Histologic analyses also revealed a natural decline in pulmonary leukocytes, with treated and untreated groups having similar counts at 30 weeks [3]. Other indicators of lung remodeling, such as radiologic density, alveolar wall thickening and collagen accumulation, were attenuated to varying degrees in all pamrevlumab treatment groups, but because these readouts tend to reflect structural rather than mRNA-producing cellular changes, they did not always align perfectly with our mRNA results. For example, lung architecture at 30 weeks was essentially normal in the 20- and 112-day treatment groups, while lung remodeling was still evident when treatment was begun 2 days before or after RT [3]. Thus, the observation that all pamrevlumab regimens attenuated the RT-induced expression pattern at 30 weeks may reflect a resolution of active fibrogenesis in the face of residual fibrosis. Either way, the attenuation of lung remodeling by all pamrevlumab regimens is consistent with the normalization of RT-induced expression changes by each of the regimens at 30 weeks.

### Changes in gene expression reveal changes in the cellular composition of lung after RT and pamrevlumab

In seeking to characterize functional relationships between altered genes, we noted changes in familiar transcripts that might be explained by changes in the relative proportions of specific cell-types. GO and GSEA do not easily distinguish between transcriptional changes that occur due to signaling within a cell as opposed to changes that reflect differences in fractional representation of infiltrating or proliferating cell-types, as occurs in injured tissue. As our attempts to identify “archetypic” mRNA markers of cell-type origin often suggested broader expression patterns, we developed a cell-type atlas approach to cope with a scarcity of objective gene panels permitting identification and quantification of broader changes in sets of diverse cell-type markers. From both the frequency of identification of cell-type distinguishing genes (Table 1) and the relative fold-change of individual cell-type distinguishing transcripts (Table 2), we conclude with high confidence that a substantial portion of transcriptional change in RT-treated lungs in our dataset arises from accumulation and pamrevlumab-induced resolution of specific cell-types; notably macrophages, dendritic, mesenchymal and mast cells. Using this approach, we characterize > 70% of the genes in the top-scoring GSEA gene-set (M4345) as likely macrophage derived [22]. Thus, these coordinated changes across cell-type distinguishing genes suggest altered recruitment, proliferation or survival of specific cell-types in RT-injured lungs.

Conclusions regarding transcripts that did not meet criteria for inclusion in cell-type distinguishing lists are mixed. For example, a clear RT-induced increase in mesenchymal markers was accompanied by a decrease in contractility markers. However, injured lungs are populated by at least three contractile cell-types of mesenchymal origin: myofibroblast, vascular smooth muscle, and bronchiolar smooth muscle cells. One interpretation is that myofibroblasts may become more numerous following RT, increasing the representation of mesenchymal markers, while bronchiolar tissue, including bronchiolar smooth muscle cells, may be partially lost, resulting in diminished contractility marker expression. Further studies would thus be warranted to characterize the more complex patterns observed in these studies.

The observations of increased representation of mast cells and other immune cell-types in this study were not highlighted in other genomic studies of similar design. To determine if these differences were related to study design or our analysis approach, we examined array data from Paun et al. concerning the effects of 18 Gy RT in three mouse strains [35]. The transcriptional response to RT in their study was very similar to ours (Additional file 7: Table S7), since 45% of genes elevated > 2-fold (*p* < 0.05) by RT in their study were also members of our RT-elevated gene-set (χ^2^ *p* <  0.0001). Genes from our cell-type lists for macrophages, mast cells, dendritic and mesenchymal cells were also highly over-represented in the Paun study (5-to-17-fold, *p* <  0.0001). Thus, the cell-type enrichments reported here are not unique to the current study, but can be revealed using the analysis methods described here.

We also examined data from Mathew et al. [36] concerning the effects of RT in lung 6 weeks after 25 Gy. Again, we observed exceptional overlap with our data (Additional file 7: Table S7), with 89% of their RT-elevated genes being members of our RT-elevated gene-set (*p* <  0.0001), including strong enrichment of macrophage genes, (*p* <  0.0001). Genes assigned by Mathew et al. to leukocyte extravasation and dendritic cell maturation, were also consistent with our results. However, at the six-week time-point used by Mathew et al., mast cell, dendritic cell and mesenchymal genes were not highly enriched. Thus RT-induced macrophage enrichment may precede enrichment or activation of other cell-types.

### An integrated model of the combined role of enriched cells and CTGF in RT-injured lung

Pamrevlumab reversed injury-associated gene signatures arising from multiple cell-types, implying interdependence of the cell-types identified. We integrated these results into a model in which RT-induced lung damage, and its resolution by CTGF inhibition, involves communication between multiple cell-types (Fig. 5); with extensive cross-talk occurring between the four cell-types (mesenchymal cells, macrophages, mast cells, and dendritic cells) enriched by RT and normalized by pamrevlumab in this study.

In Fig. 5, pamrevlumab inhibits CTGF elicited by mesenchymal cells in RT-injured tissue. This is inferred by extensive studies indicating that CTGF is synthesized by and influences the behavior of mesenchymal cells in culture and in fibrotic tissue [34]. In the current study, pamrevlumab also affected multiple immune cell types, suggesting control over these other cell types mediated by mesenchymal cells and CTGF. In our model, CTGF inhibition disrupts signaling pathways between multiple RT-enriched cell types, thereby attenuating RT-induced pneumonitis and fibrogenic responses. Indeed, many of the mediators identified in the current study have been associated with the reprogramming of immune responses from an inflammatory and pro-fibrotic state to a reparative response [37, 38].

Of the various mesenchymal genes in the RT-responsive gene-set, mesenchymal cell-derived CXCL12 is a candidate factor that may regulate recruitment of the immune cell-types identified as being enriched by RT [26–28]. CXCL12 promotes pulmonary fibrosis by recruiting “fibrocytes” and bone marrow-derived stem cells to injured lung [29, 39]. Mesenchymal stem cell recruitment to RT-injured lung also protects against pneumonitis and fibrosis in association with diminished induction of pulmonary CTGF by RT [40].

Our data also suggest an important role of mast cells in the cellular cross-talk in RT-injured lung. Mast cell enrichments have been reported in RT-induced lung injury models [41–43], yet their role in RT injury remains unclear, with opposing effects reported in other irradiated organs [44–47]. It has also been suggested that mast cell proteases regulate cytokines, growth factors, other proteinases and ECM remodeling in IPF [48]. Mast cell-derived IL4 is another probable component of communication between RT-enriched cell types, and as indicated in our model, it can influence each of the enriched cell types identified. IL4 is known to exert pro-fibrotic effects in lung via activation of M2 macrophages [49, 50]. IL4 also promotes fibroblast proliferation [51, 52], alters the activity of dendritic cells [53], and elicits a “mature” mast cell phenotype [54].

Macrophages enjoy a prominent role in our model, as an important regulator of fibrosis and activator of other immune cell-types and fibroblasts [55]. For example, in response to mast cell IL4, macrophages express IGF1, which promotes myofibroblast proliferation and survival [32]. In addition, macrophage-derived CCL3 regulates IL4 in RT-injured lungs [56] and mast cell IL4 can elicit macrophage RETNLA, which in turn stimulates myofibroblast activation in injured lung [57].

Our model also includes dendritic cells, common to IPF and other human fibrotic lung diseases [28]. Dendritic cells, along with other antigen-presenting cells, regulate decisions on whether to mount adaptive or innate immune responses [58]. Since RT did not result in enrichment of cells conferring adaptive immunity, our model suggests that enhanced immune surveillance may be a feature of RT-treated lung tissue. Other regulators identified in this study may be shared by the various cell-types, including *Ccl2* and *Ccl12*, which are known to influence lung fibrosis in rodents [8, 59].

Pamrevlumab has also been found to downregulate pro-survival factors XIAP and BIRC6 in a model of pancreatic cancer [60] and the related inhibitor of apoptosis BIRC5 was among the RT-elevated, pamrevlumab-diminished genes identified in the current model (Additional file 3: Table S3). Moreover, the enhanced persistence and apoptosis-resistant phenotype of myofibroblasts in IPF and other fibrotic conditions may be a consequence of anti-apoptotic factors [61]. Thus by altering the lung microenvironment, pamrevlumab may downregulate pro-survival signals in myofibroblasts, thereby suppressing fibrotic and inflammatory cell responses in RT injury and IPF.

Interpretation of down-regulated genes was less straightforward. Some decreases may result from proportional changes in cell-type representation due to displacement or degradation of normal lung tissue. For example, expression of Bmp6, a likely endothelial transcript, was strongly diminished by RT in this study and others [35]. Although this could represent a reduced contribution of alveolar capillaries to the total tissue content of injured lung, its transcriptional down-regulation seems more likely, since other endothelial genes were modestly elevated. Moreover, BMP6 function has been linked to CTGF and its genetic absence has been associated with enhanced kidney fibrosis and increased CTGF expression [62].

## Conclusions

Thoracic RT results in pneumonitis. Thus, the immune cell signatures evident in our study are no surprise. Notably, however, pamrevlumab averted or attenuated this immune response. Given the wealth of evidence that CTGF regulates mesenchymal and epithelial cell behavior, the observation that CTGF inhibition affects immune cell responses in vivo suggests that these effects occur via mesenchymal intermediates. Thus CTGF appears to be intimately involved in complex cellular cross-talk following RT, contributing simultaneously to immune and fibrotic responses, since both components were attenuated by pamrevlumab. Precisely how CTGF orchestrates these interactions, however, will require further study.

## Additional files

Additional file 1: Table S1. Array Files used to Assemble a Human Cell-Type Expression Atlas and Normailzed CTGF Expression. (XLSX 20 kb)

Additional file 2: Table S2. Cell-Type Distinguishing Gene Lists. (XLSX 59 kb)

Additional file 3: Table S3. Radiation and FG-3019 Responsive Genes (*n* = 2754). (XLSX 674 kb)

Additional file 4: Table S4. GO categories associated with the RT-elevated gene set. (XLSX 24 kb)

Additional file 5; Table S5. GO categories associated with the RT-diminished gene set. (XLSX 12 kb)

Additional file 6: Table S6. Broad Gene Sets Associated with Radiation and FG-3019 Responsive Genes. (XLSX 18 kb)

Additional file 7: Table S7. Enrichment of RT and FG-3019 Responsive Genes and Cell-Type Distinguishing Genes in Other Public Lung RT Gene Sets. (XLSX 19 kb)

## Abbreviations

ANKRD34B: Ankyrin repeat domain 34B
ARG1: Arginase 1
BIRC5: Baculoviral IAP repeat containing 5
BIRC6: Baculoviral IAP repeat containing 6
BMP6: Bone morphogenetic protein 6
CCL12: C-C motif chemokine ligand 12
CCL17: C-C motif chemokine ligand 17
CCL2: C-C motif chemokine ligand 2
CCL3: C-C motif chemokine ligand 3
CCL4: C-C motif chemokine ligand 4
CCL5: C-C motif chemokine ligand 5
CCL7: C-C motif chemokine ligand 7
CHI3L3: Chitinase 3-like 3
CHI3L4: Chitinase 3-like 4
CSF2RB: Colony stimulating factor 2 receptor beta
CT: Computed tomography
CTGF: Connective tissue growth factor
CXCL10: C-X-C motif chemokine ligand 10
CXCL12: C-X-C motif chemokine ligand 12 (stromal-derived factor 1)
CXCL9: C-X-C motif chemokine ligand 9
ECM: Extracellular matrix
FCER1G: Fc receptor, IgE, high affinity I, gamma polypeptide
FGF2: Fibroblast growth factor 2
GO: Gene ontology
GSEA: Gene-set enrichment analysis
IGF1: Insulin-like growth factor 1
IGFBP4: Insulin-like growth factor binding protein 4
IgG: Immunoglobulin G
IL12A: Interleukin 12A
IL16: Interleukin 16
IL18: Interleukin 18
IL1B: Interleukin 1 beta
IL1RN: Interleukin 1 receptor antagonist
IL4: Interleukin 4
IL6: Interleukin 6
INHBA: Inhibin beta-A
IPF: Idiopathic pulmonary fibrosis
MMP12: Matrix metallopeptidase 12
MRC1: Mannose receptor C-type 1
NOS2: Nitric oxide synthase 2
NPY: Neuropeptide Y
PCA: principle component analysis
RETNLA: Resistin-like alpha
RT: Radiation or Radiation Treatment
SPP1: Secreted Phosphoprotein 1, (osteopontin)
TGFβ: Transforming growth factor beta
TIW: Three times a week
TNF: Tumor necrosis factor
XIAP: X-linked inhibitor of apoptosis
